# Work stress and loss of years lived without chronic disease: an 18-year follow-up of 1.5 million employees in Denmark

**DOI:** 10.1007/s10654-022-00852-x

**Published:** 2022-03-21

**Authors:** Jeppe K. Sørensen, Elisabeth Framke, Jacob Pedersen, Kristina Alexanderson, Jens P. Bonde, Kristin Farrants, Esben M. Flachs, Linda L. Magnusson Hanson, Solja T. Nyberg, Mika Kivimäki, Ida E. H. Madsen, Reiner Rugulies

**Affiliations:** 1 National Research Centre for the Working Environment, Lersø Parkalle 105, 2100 Copenhagen, Denmark; 2 Division of Insurance Medicine, Department of Clinical Neuroscience, Karolinska Institutet, 171 77 Stockholm, Sweden; 3 Department of Occupational and Environmental Medicine, Bispebjerg and Frederiksberg Hospital, Bispebjerg Bakke 23F, 2400 Copenhagen, Frederiksberg, Denmark; 4 Department of Public Health, University of Copenhagen, Øster Farimagsgade 5, 1014 Copenhagen, Denmark; 5 Stress Research Institute at Department of Psychology, Stockholm University, Frescati Hagväg 16A, 114 19 Stockholm, Sweden; 6 Department of Public Health, Clinicum, Faculty of Medicine, University of Helsinki, Yliopistonkatu 3, 00014 Helsinki, Finland; 7 Finnish Institute of Occupational Health, Topeliuksenkatu 41 B, 00250 Helsinki, Finland; 8 Department of Epidemiology and Public Health, University College of London, 1-19 Torrington Place, London, WC1E 6BT UK; 9 Department of Psychology, University of Copenhagen, Øster Farimagsgade 2A, 1353 Copenhagen, Denmark; 10 The Danish Multiple Sclerosis Registry, Copenhagen University Hospital, Blegdamsvej 9, 2100 Copenhagen, Denmark

**Keywords:** Work stress, Job strain, Effort-reward imbalance, Life expectancy, Chronic disease, Register-based research

## Abstract

We aimed to examine the association between exposure to work stress and chronic disease incidence and loss of chronic disease-free life years in the Danish workforce. The study population included 1,592,491 employees, aged 30–59 in 2000 and without prevalent chronic diseases. We assessed work stress as the combination of job strain and effort-reward imbalance using job exposure matrices. We used Cox regressions to estimate risk of incident hospital-diagnoses or death of chronic diseases (i.e., type 2 diabetes, coronary heart disease, stroke, cancer, asthma, chronic obstructive pulmonary disease, heart failure, and dementia) during 18 years of follow-up and calculated corresponding chronic disease-free life expectancy from age 30 to age 75. Individuals working in occupations with high prevalence of work stress had a higher risk of incident chronic disease compared to those in occupations with low prevalence of work stress (women: HR 1.04 (95% CI 1.02–1.05), men: HR 1.12 (95% CI 1.11–1.14)). The corresponding loss in chronic disease-free life expectancy was 0.25 (95% CI − 0.10 to 0.60) and 0.84 (95% CI 0.56–1.11) years in women and men, respectively. Additional adjustment for health behaviours attenuated these associations among men. We conclude that men working in high-stress occupations have a small loss of years lived without chronic disease compared to men working in low-stress occupations. This finding appeared to be partially attributable to harmful health behaviours. In women, high work stress indicated a very small and statistically non-significant loss of years lived without chronic disease.

**Supplementary Information:**

The online version contains supplementary material available at 10.1007/s10654-022-00852-x.

## Introduction

In the past three decades, the global average life expectancy has increased markedly [1, 2]. In high income countries, such as Denmark, the average life expectancy at birth has on average increased by 4.9 years in women and 6.6 years in men from 1990 to 2017 with approximately 14% of the life expectancy from birth lived in poor health [2]. Non-communicable diseases such as coronary heart disease (CHD), low back pain, stroke, lung cancer, and chronic obstructive pulmonary disease (COPD) are some of the leading causes of years lived with morbidity in high-income countries [2].

Growing evidence suggests that psychosocial work stress is associated with a higher risk of various health problems. Work stress conceptualised according to two theoretical models, the job strain model [3] (the combination of high job demands and low job control) and the effort-reward imbalance (ERI) model [4] (the combination of high efforts and low rewards, in terms of salary, appreciation, job security and promotion prospects), have previously predicted incident chronic diseases such as CHD [5–7], stroke [8], and diabetes [9, 10] in large-scale cohort studies. An additive effect of job strain and ERI have previously been established in the risk of CHD [7].

To our knowledge, only one study has assessed the potential contribution of work stress on chronic disease-free life expectancy [11]. With pooled data from 64,832 employees from Finland, France, Sweden, and the United Kingdom, the study reported that women and men with job strain had 0.6 and 0.8 fewer years without chronic diseases from age 50 to 75, respectively, compared to those without job strain [11]. Disease-free life expectancy before age 50 or an effect of effort-reward imbalance, in addition to job strain, were not considered in that study. Further, of the four cohorts, two cohorts included public sector employees, only, and one cohort included workers from a single employer, only.

In the present study, we examined whether work stress, measured as the combination of job strain and ERI, is associated with risk of incident chronic disease and chronic disease-free life expectancy from age 30 to 75 in the Danish workforce. With detailed information from nationwide registers and job exposure matrices (JEM), we followed a cohort of more than 1.5 million employees, aged 30–59 and without chronic diseases at baseline, for 18 years. We defined chronic disease according to the World Health Organisation’s priority of non-communicable chronic diseases target for prevention [1, 2] (type 2 diabetes, CHD, stroke, cancer, asthma, COPD) and further added heart failure and dementia as in a recent study [12].

## Methods

### Study design and population

We used data from the JEMPAD (Job Exposure Matrix Analyses of Psychosocial Factors and Healthy Ageing in Denmark) cohort, a Danish population-based cohort with information on employment, psychosocial factors at work, health, and socio-demographics [6, 13]. The study population was drawn from the Integrated Database for Labour Market Research (IDA) at Statistics Denmark [14] and consisted of all individuals residing in Denmark in the year 2000, aged 30–59, who were employed (excluding the self-employed), and had complete data on age, sex, and migration background yielding 1,680,214 individuals. Using the individuals’ unique Danish civil registration number, we linked these individuals to other population-based registers providing individual-level information on socio-demographics, use of health services, diagnoses for hospital treatment (in- and outpatient), and causes of death.

**Fig. 1:**
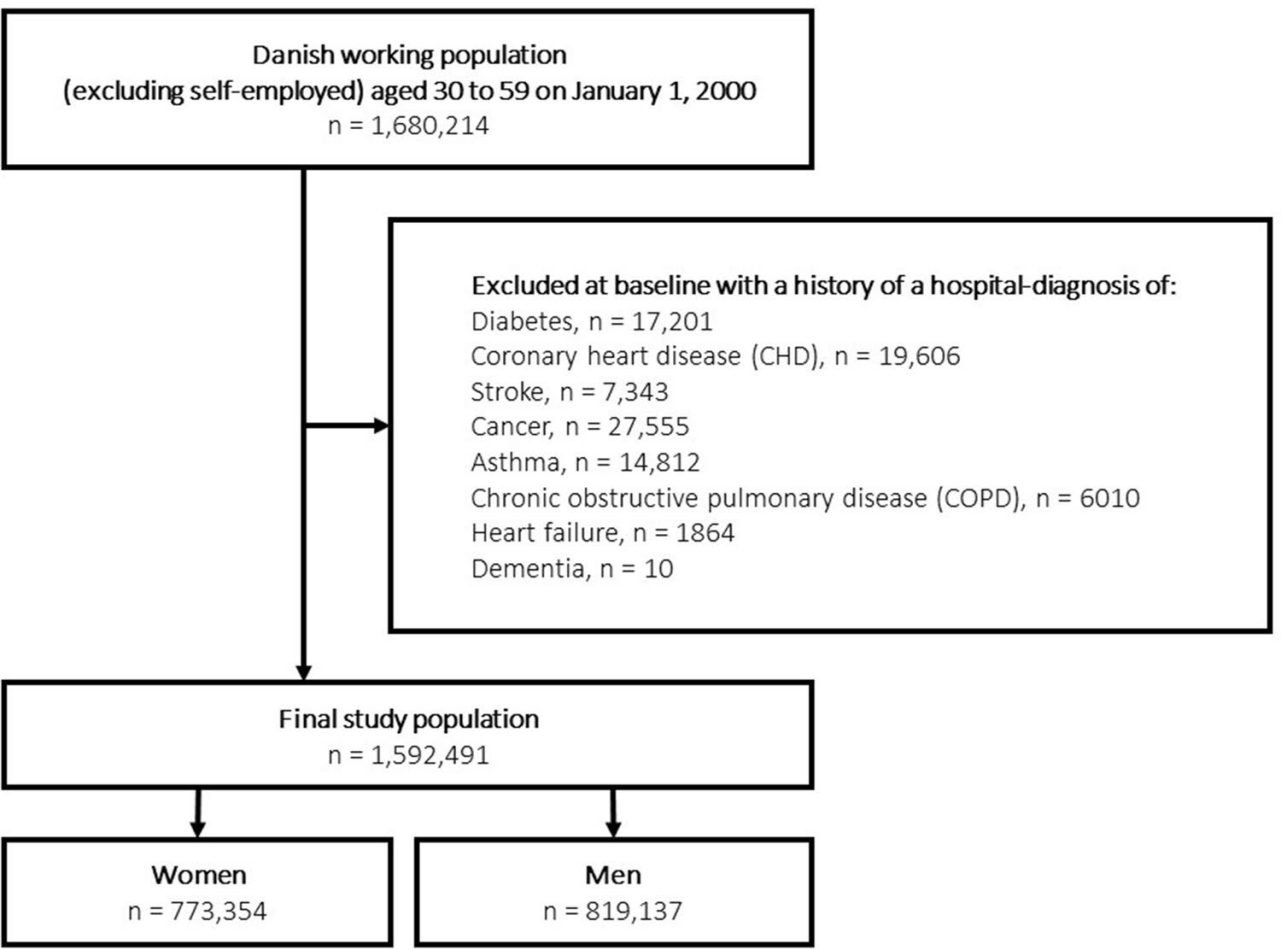
Flowchart of the final study population

We included individuals without a history of a hospital-diagnosis of any of eight chronic diseases (diabetes (type 1 or type 2), CHD, stroke, cancer, asthma, COPD, hearth failure, and dementia). We excluded 87,723 (5.5%) individuals with one or more of these diseases [diabetes (n = 17,201), CHD (19,606), stroke (7343), cancer (27,555), asthma (14,812), COPD (6010), heart failure (1864), and dementia (10)] diagnosed from 1977 (when information on diagnosed diseases became available in the registers) to 31 December 2000 (study baseline). The final study population consisted of 1,592,491  individuals (773,354 women and 819,137 men). Figure 1 presents a flowchart for the study population. As data was linked with population-based registers, none of the cohort members were lost to follow-up. Participants who emigrated from Denmark or who died of other causes than from the diseases under study, were censored at the date of emigration and death, respectively.

To estimate the number of years without any of the eight chronic diseases we linked the study population with individual records from the same national registers until the end of follow-up (31 December 2018).

### Work stress

We estimated work stress as the combination of job strain and ERI using JEMs based on information from the Danish Work Environment Cohort study (DWECS) [15, 16].

In DWECS, job strain was measured using three items on psychological demands at work and five items on job control from DWECS. In accordance with previous research [5, 8, 13, 17], we defined job strain as higher than the median on score for psychological demands and lower than the median on the score for job control. In line with previous research [7, 18], we defined ERI in DWECS as the combination of four items on effort and four items on reward and calculated an effort-reward ratio and defined respondents with an effort-reward ratio above one as having ERI. Area under the curve (AUC) for the JEMs was 0.70 and 0.73 for job strain and ERI, respectively. Supplementary material, Appendix 1, including Table A1, provides a detailed description of the measurement of job strain and ERI and the construction of the JEMs. We assigned the predicted probability of job strain and ERI, respectively, to each individual in the JEMPAD cohort by job group, sex, and age in 2000. We categorised each cohort member into high and low prevalence of job strain and ERI based on previous results on the overall prevalence of job strain and ERI from a pooled European study of 90,164 participants conducted between 1985 and 2005 in Denmark, Finland, France, Germany, Sweden, and the United Kingdom (the “IPD-Work consortium”) [7]. The pooled prevalence from the 11 studies were 15.9% and 31.7% for job strain and ERI, respectively [7]. We applied this information on the pooled prevalence to JEMPAD by categorizing the top 15.9% and the top 31.7% of the cohort as high prevalence of job strain and ERI, respectively. We defined work stress as a joint work stress variable of exposure to job strain and ERI simultaneously. We categorised individuals into four groups: (1) no stressors (not exposed to job strain and ERI), (2) job strain only (exposed to job strain but not ERI), (3) ERI only (exposed to ERI but not job strain), and (4) both stressors (exposed to both job strain and ERI). In the groups of individuals categorised as exposed to both stressors, the majority were employed in elementary occupations (37.5%) such as cleaners and helpers, food preparation and manufacturing, and as general office clerks (23.9%).

### Chronic disease outcome

We defined chronic diseases based on the World Health Organisation’s priority of non-communicable chronic diseases target for prevention including type 2 diabetes, CHD, stroke, cancer, asthma, and COPD [1, 2] and further added heart failure and dementia as suggested by Nyberg et al. [12] We ascertained incident chronic disease by diagnoses from the National Patient Register [19] (including both main and secondary diagnoses) and the Danish Register of Causes of Death [20] (including both underlying and contributing causes) from 1 January 2001 to 31 December 2018.

We defined the eight chronic diseases by hospital-diagnosis or death during follow-up with ICD-10 codes (see Supplementary material, Appendix 2). We defined prevalent chronic diseases by hospital-diagnosed chronic diseases during or before the baseline year with ICD-8 and ICD-10 codes (ICD-9 was never used in Denmark) from 1977 (outpatient data available from 1995) to 31 December 2000 (see Supplementary material, Appendix 3).

### Covariates

From population-based registers [21–24] we included sex (women and men), age, migration background (Danish origin (the whole population in Denmark except immigrants and descendants of immigrants), immigrants (born abroad and none of the parents were either Danish citizens or born in Denmark), and descendants of immigrants (born in Denmark and none of the parents were either Danish citizens or born in Denmark)), family type (single without children, single with children below age 8, single with children age 8–17 without children below age 8, married/cohabitant without children, married/cohabitant with children below age 8, or married/cohabitant with children age 8–17 without children below age 8) as covariates. We further included health service use as an indicator for health status (measured as the number of yearly contacts and services within the primary health care system in quartiles) and socioeconomic position (measured by equivalent household disposable income accounting for household size in quartiles) as they might be associated with both work stress and risk of incident chronic disease.

We further included number of risky health behaviours (risk of smoking, high weekly alcohol intake, high BMI, and low leisure time physical activity) estimated by job group aggregated JEMs from the Danish Occupational Cohort (DOC*X) study [25] as potential confounders or mediators. Intraclass correlation coefficients were 3.52%, 2.12%, 2.81%, and 0.26% for smoking, BMI, alcohol, and leisure time physical activity, respectively. We calculated the number of risky health behaviours separately for women and men to account for overall sex differences in the JEMs. Based on the distributions of the predicted probability of smoking, predicted level of BMI, and the predicted level of weekly alcohol consumptions, we categorised individuals into high risk of smoking, high BMI, and high weekly alcohol consumption with cut-points at the highest tertile, respectively. Based on the distribution of the predicted level of leisure time physical activity, we categorised individuals into low leisure time physical activity with a cut-point at the lowest tertile. These cut-off points correspond at the occupational level to a predicted probability of smoking of 30% or higher, a weekly alcohol intake of more than 7 units/week, a predicted level of BMI higher than 25, and leisure time physical activity of less than 2 h. The number of risky health behaviours was calculated for each individual and as few individuals were assigned four risky health behaviours, we collapsed three and four risky health behaviours.

We measured all covariates in 2000 except the number of health services used, which we measured one year before baseline (1999) to ensure that use of health services took place before the measurement of work stress. See Supplementary material, Appendix 4, for a detailed description of the covariates.

### Statistical analysis

All analyses were conducted in SAS 9.4 separately for women and men to account for overall sex-differences in the average chronic disease-free life expectancy in Denmark [26] and overall sex-segregation of the Danish labour market [27]. Using Cox proportional hazard model we estimated the hazard ratio (HR) and 95% confidence intervals (CI) for the risk of incident chronic disease using the PHREG procedure. We defined age as the underlying timescale from 1 January 2001 until the first event or censuring due to migration, death (due to other reasons than the eight chronic diseases under study), or end of follow-up, 31 December 2018, whichever came first. We calculated crude associations as cases per 1000 person years, and conducted crude survival analyses with age as the underlying time scale (model 1) and analyses further adjusted for migration background, family type, number of health services used, and household disposable income (model 2), as the main model of the analysis. In addition, we computed a model further adjusted for the number of risky health behaviours (model 3). We considered this model as over-adjusted, as risky health behaviours are likely not only confounders but also potential important intermediate steps in the pathway linking exposure to work stressors with incident chronic disease [28–30]. Consequently, we did not consider model 3 as the main model, but we still wanted to conduct this model, as this could provide insight into possible mechanisms between work stress and chronic disease-free life expectancy [12].

Based on the baseline function from the Cox proportional hazard models we estimated the chronic disease-free life expectancy by calculating the estimated mean survival time from age 30 to age 75 as the area under the estimated survival curve for all possible combinations of work stress and covariates. We then assigned the mean survival time to all individuals based on their individual covariate structure. We estimated 95% confidence intervals for the mean differences in chronic disease-free life expectancy using the 95% upper and lower confidence limit of the estimated survival curves from the baseline function as previously suggested [31, 32]. We defined statistically significant differences in chronic disease-free life years lost due to work stress as non-overlapping confidence intervals.

### Supplementary analysis

All supplementary analyses were adjusted for the covariates in model 2. First, we performed an analysis restricted to the six non-communicable chronic diseases priorities by WHO as target for prevention (type 2 diabetes, CHD, stroke, cancer, asthma, COPD) [1, 2]. Second, we conducted an analysis on exposure contrast by using the DWECS 2000 specific prevalence of job strain and ERI instead of the pooled prevalence’s retrieved from the IPD-Work consortium (job strain: 10.7% instead of 15.9% and ERI: 23.8% instead of 31.7%) [7]. Third, we estimated the association between work stress and incident chronic disease and chronic disease-free life expectancy from age 50 to 75 in a subsample of individuals age 50 or above at baseline (n = 461,141). Fourth, we estimated the incidence of chronic diseases in subgroups of household disposable income in quartiles (low, medium–low, medium–high, and high). Fifth, we conducted outcome-specific analyses for the association between work stress and incident risk of the eight included chronic diseases separately. We grouped the eight chronic diseases as described in Supplementary material, Appendix 2 and censored due to hospital-diagnosis or death due to another chronic disease. Sixth, we analysed job strain and ERI as separate exposures. Finally, we estimated age and sex-adjusted associations between the covariates and incident risk of chronic diseases and chronic disease-free life expectancy.

## Results

### Population characteristics

The prevalence of sociodemographic and health characteristics for the 773,354 women and 819,137 men at baseline are presented in Table 1. The mean age in both sexes was 44 and most individuals had no migration background (women = 95.9% and men = 95.1%) and were married or cohabitant (women = 64.7% and men = 63.2%).

**Table 1:** Sociodemographic characteristics, health services used, health behaviours and work stress among women and men at baseline

	Women n = 773,354		Men n = 819,137	
*Sociodemographics*				
Age, mean (SD)	44	(8.3)	44	(8.4)
* Migration background*				
No migration background, n (%)	741,339	(95.9)	77,098	(95.1)
Immigrant, n (%)	30,901	(4.0)	38,785	(4.7)
Descendant of immigrants, n (%)	1114	(0.1)	1254	(0.2)
* Family type*				
Single without children, n (%)	120,296	(15.6)	185,686	(22.7)
Single with children (age 0–7), n (%)	18,010	(2.3)	8870	(1.1)
Single with children (age 8–17), n (%)	40,089	(5.2)	14,877	(1.8)
Married/cohabitant without children, n (%)	244,212	(31.6)	240,039	(29.3)
Married/cohabitant with children (age 0–7), n (%)	106,419	(13.8)	131,844	(16.1)
Married/cohabitant with children (age 8–17), n (%)	149,386	(19.3)	146,187	(17.8)
Unknown family type, n (%)	94,942	(12.3)	91,634	(11.2)
Yearly equivalent household disposable income*				
EUR, mean (SD)	42,490	(39 958)	41,222	(31 455)
GBP, mean (SD)	36,784	(34 592)	35,687	(27 231)
USD, mean (SD)	50,013	(47 033)	48,521	(37 024)
*Health services used*				
Yearly health services used, mean (SD)*	19	(21.3)	11	(15.4)
*Health behaviours*				
* Number of risky health behaviours*				
No risky health behaviours, n (%)	111,103	(14.4)	144,624	(17.7)
One risky health behaviours, n (%)	334,294	(43.2)	344,927	(42.1)
Two risky health behaviours, n (%)	181,218	(23.4)	108,749	(13.3)
Three and four risky health behaviours, n (%)	79,714	(10.3)	143,264	(17.5)
Unknown number of health behaviours, n (%)	67,025	(8.7)	77,573	(9.5)
*Work stress*				
* Number of work stressors*				
No stressors, n (%)	485,263	(62.7)	485,422	(59.3)
Job strain only, n (%)	60,793	(7.9)	208,330	(25.4)
ERI only, n (%)	161,155	(20.8)	55,432	(6.8)
Both stressors, n (%)	66,143	(8.6)	69,953	(8.5)
* Job strain*				
Low prevalence, n (%)	646,418	(83.6)	693,752	(84.7)
High prevalence, n (%)	126,936	(16.4)	125,385	(15.3)
* Effort-reward imbalance*				
Low prevalence, n (%)	546,056	(70.6)	540,854	(66.0)
High prevealence, n (%)	227,298	(29.4)	278,283	(34.0)

*These skewed variables were divided into quartiles and treated as categorical variables in the analyses

### Incident chronic disease and disease-free life years lost

Among women, during 12,283,478 person-years at risk, we identified 176,319 (22.8%) cases of incident chronic disease [type 2 diabetes: 2.6% (n = 19,909), CHD: 1.1% (n = 8230), stroke: 2.4% (n = 18,296), cancer: 11.5% (n = 88,871), asthma: 2.4% (n = 18,929), COPD: 2.2% (n = 17,107), heart failure: 0.6% (n = 4280), and dementia: 0.1% (n = 697)]. Among men, during 12,608,153 person-years at risk, we identified 215,359 (26.3%) cases of incident chronic disease (type 2 diabetes (n = 34,002): 4.2%, CHD: 3.5% (n = 28,281), stroke: 3.5% (n = 28,978), cancer: 9.8% (n = 80,351), asthma: 1.6% (n = 12,930), COPD: 2.2% (n = 17,919), heart failure: 1.5% (n = 12,105), and dementia: 0.1% (n = 793)].

**Fig. 2:**
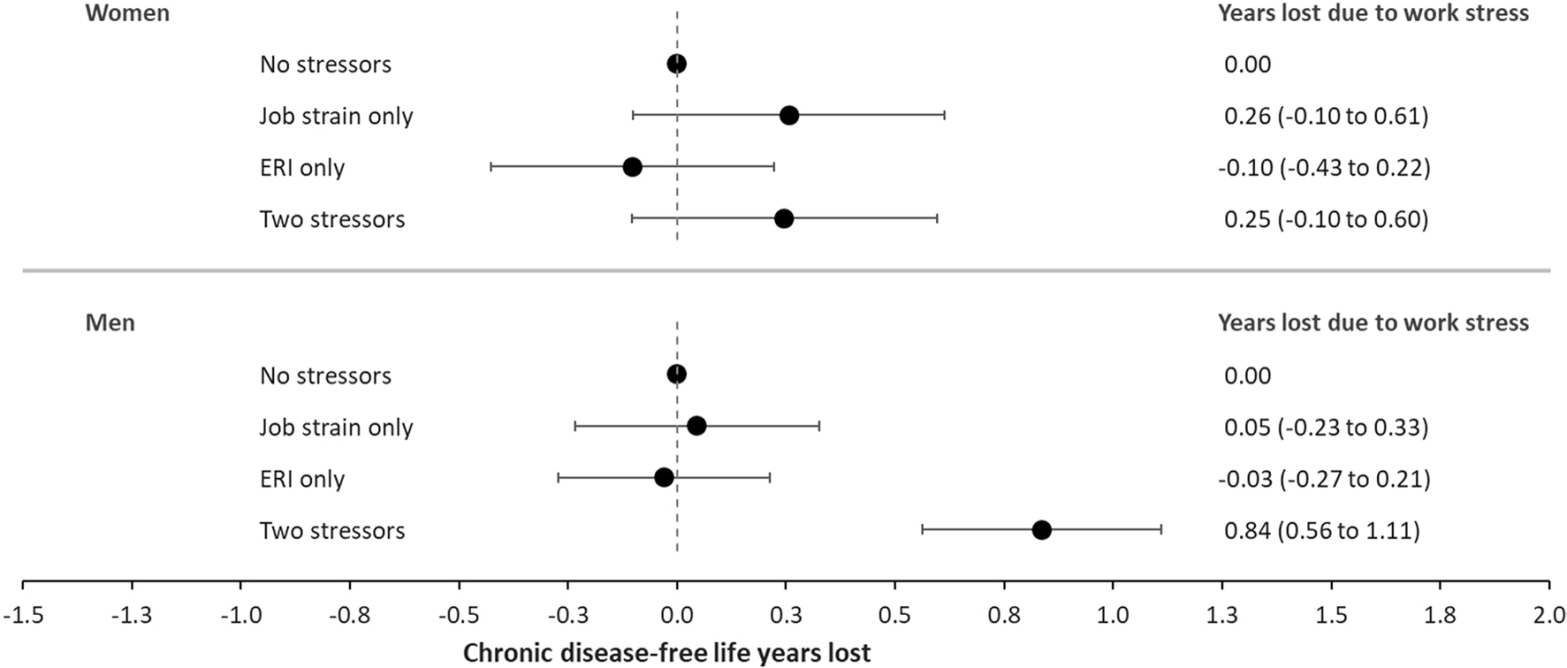
Years lost of chronic disease-free life years among women (n = 773,355) and men (n = 819,138) by exposure to work stressors. Adjusted for covariates of model 2. Years lost of chronic disease-free life years from age 30 to 75 adjusted for age (underlying time scale), migration background, family type, household disposable income, and number of health services used

Table 2 shows cases per 1000 person-years and HR and 95% CI for the age-adjusted (model 1) and multivariable adjusted (model 2) risk of incident chronic disease and multivariable adjusted chronic disease-free life expectancy from age 30 to 75 among women and men separately. The corresponding chronic disease-free life years lost due to work stress are presented in Fig. 2.

**Table 2:** Association between work stress and incident chronic disease and chronic disease-free life expectancy from age 30 to 75 among women and men

	Person-years	Cases	Cases per 1000 person-years	Hazard ratio (95% CI) for incident chronic disease		Chronic disease-free life expectancy (95% CI)***
				Model 1*	Model 2**	
Women (n = 773,355)	12,283,478	176,319	14.4			
* Work stressors*						
No stressors	7,771,304	101,794	13.1	1.00	1.00	37.3 (37.1–37.4)
Job strain only	979,386	12,740	13.0	1.04 (1.02–1.05)	1.04 (1.02–1.06)	37.0 (36.8–37.2)
ERI only	2,491,304	44,871	18.0	0.98 (0.97–0.99)	0.99 (0.97–1.00)	37.4 (37.2–37.5)
Both stressors	1,041,483	16,914	16.2	1.04 (1.03–1.06)	1.04 (1.02–1.05)	37.0 (36.8–37.2)
Men (n = 819,138)	12,608 153	215,355	17.1			
* Work stressors*						
No stressors	7,592,270	111,020	14.6	1.00	1.00	36.5 (36.4–36.6)
Job strain only	875,554	12,829	14.7	1.01 (1.00–1.03)	1.01 (0.99–1.03)	36.4 (36.3–36.6)
ERI only	3,087,824	69,354	22.5	0.99 (0.98–1.00)	1.00 (0.99–1.01)	36.5 (36.4–36.6)
Both stressors	1,052,505	22,152	21.0	1.17 (1.15–1.18)	1.12 (1.11–1.14)	35.6 (35.5–35.8)

*Adjusted for age (underlying time scale)

**Adjusted for age (underlying time scale), migration background, family type, household income and number of health services used

***Estimated life years free from chronic disease from age 30 to 75 adjusted for age (underlying time scale), migration background, family type, household income and number of health services used

Compared to individuals with low prevalence of work stress, exposure to high prevalence of work stress (job strain and ERI simultaneous) was associated with increased risk of chronic diseases among both women and men with HRs of 1.04 (95% CI 1.02–1.05, women) and 1.12 (95% CI 1.11–1.14, men), respectively (Table 2, model 2). Hazard ratios of exposure to both work stressors corresponded to 0.25 (95% CI − 0.10 to 0.60, women) and 0.84 (95% CI 0.56–1.11, men) fewer years free from chronic diseases from age 30 to 75 when exposed to both work stressors compared to not being exposed (Fig. 2). Among women, the association between work stressors and loss of years lived without chronic disease was driven by job strain. The confidence intervals of all estimates included unity. Among men, neither job strain only nor ERI only were associated with loss of years lived without chronic disease. However, the simultaneous presence of both job strain and ERI was associated with a reduction in years lived without chronic disease, with a confidence interval not including unity (Fig. 2).

### Adjusting for risky health behaviours

**Fig. 3:**
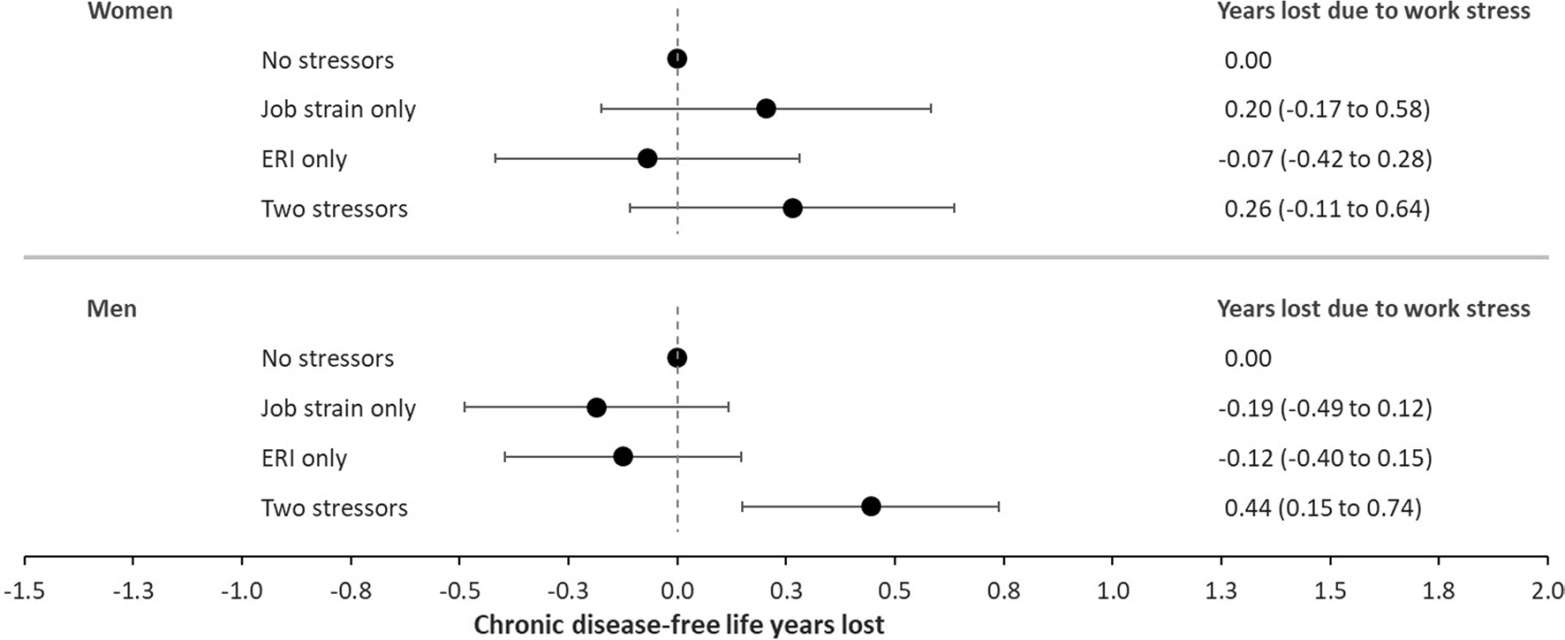
Years lost of chronic disease-free life years among women (n = 773,355) and men (n = 819,138) by exposure to work stressors. Adjusted for covariates of model 3. Years lost of chronic disease-free life years from age 30 to 75 adjusted for age (underlying time scale), migration background, family type, household disposable income, number of health services used and number of risky health behaviours

Table 3 shows the association between the work stressors and incident chronic disease after further adjustment for health behaviours, which may be a mechanism linking work stressors to chronic disease. Compared to the main analysis (Table 2), estimates virtually remained unchanged among women and were attenuated among men (Table 3). Consequently, loss of chronic disease-free life years after adjustment for risky health behaviours (Fig. 3), compared to the main analysis were similar among women (0.26 vs. 0.25) and lower among men (0.44 vs. 0.84).

**Table 3:** Association between work stress and incident chronic disease and chronic disease-free life expectancy from age 30 to 75 among women and men adjusted for number of risky health behaviours

	Hazard ratio (95% CI) for incident chronic disease	Chronic disease-free life expectancy (95% CI)*
	Model 3*	
Women (n = 773,355)		
* Work stressors*		
No stressors	1.00	37.7 (37.5–37.8)
Job strain only	1.03 (1.01–1.05)	37.5 (37.3–37.7)
ERI only	0.99 (0.98–1.00)	37.7 (37.6–37.9)
Both stressors	1.04 (1.02–1.06)	37.4 (37.2–37.6)
Men (n = 819,138)		
* Work stressors*		
No stressors	1.00	36.6 (36.4–36.7)
Job strain only	0.98 (0.96–0.99)	36.7 (36.6–36.9)
ERI only	0.98 (0.97–0.99)	36.7 (36.6–36.8)
Both stressors	1.06 (1.05–1.08)	36.1 (36.0–36.3)

*Incident risk of chronic diseases and estimated life years free from chronic disease from age 30 to 75 adjusted for age (underlying time scale), migration background, family type, household disposable income, number of health services used, and number of risky health behaviours

### Supplementary analysis

When repeating the main analysis while restricting the outcome to the six non-communicable chronic diseases priorities by WHO as target for prevention we found similar results (Supplementary material, Appendix-5, Table A-2). Increasing exposure contrast using a higher cut off point for job strain and ERI, yielded stronger association among women (HR 1.06, 95% CI 1.04–1.08, years lost due to work stress 0.41, 95% CI 0.03–0.79) and similar results among men compared to the main analysis (Supplementary material, Appendix-5, Table A-3). Among individuals age 50 or above at baseline, we found similar results as in the main analysis (Supplementary material, Appendix-5, Table A-4). When we analysed job strain and ERI not in combination but separately, we found among women that job strain (HR 1.04, 95% CI 1.03–1.06) but not ERI (HR 1.00, 95% CI 0.99–1.01) was associated with increased risk of incident chronic disease. Among men, both job strain (HR 1.08, 95% CI 1.07–1.10) and ERI (HR 1.02, 95% CI 1.02–1.03) were associated with increased risk of incident chronic disease (Supplementary material, Appendix-5, Table A-5). When we analysed the association between work stress and incident chronic disease by sub-groups of household disposable income, we found similar associations among men in all quartiles. Among women, associations were slightly stronger in the medium–low and medium–high quartile than in the low and the high quartile (Supplementary material, Appendix-5, Table A-6). The outcome-specific analyses showed among women significant higher risk in two out of eight chronic diseases (CHD and COPD) and among men in six out of eight chronic diseases (type 2 diabetes, CHD, stroke, cancers, COPD and heart failure) (Supplementary material, Appendix-5, Table A-7). In none of the outcome-specific analyses was work stress associated with lower risk of a chronic disease. Finally, we examined the association between the covariates (sociodemographic characteristics, number of health services used and number of risky health behaviours) and risk of incident chronic disease and chronic disease-free life expectancy (Supplementary material table, Appendix-5, Table A-8). We found an increased risk of incident chronic disease and fewer chronic disease-free life years among men compared to women, immigrants compared to those with no migration background, singles without children compared to all other family types, individuals with low household income compared to those with high household income, individuals with a higher number of health service used compared to those with low number of health services used, and individuals with two, three or four risky health behaviours compared to those with no risky health behaviours.

## Discussion

In this study of more than 1.5 million employees in the Danish workforce, occupational level work stress, measured with JEMs as the combination of job strain and ERI, was associated with a higher risk of incident chronic disease defined as hospital-diagnosis or death due to type 2 diabetes, CHD, stroke, cancer, asthma, COPD, hearth failure, or dementia. The association with incident chronic diseases was present among both women and men with excess risk of 1.04 and 1.12, respectively in the main model. Among women, high prevalence of work stress was inconclusively associated with a loss of chronic disease-free life expectancy of 0.25 years among women, with confidence intervals including unity. Among men, high prevalence of work stress was associated with a statistical significant loss of chronic disease-free life expectancy of 0.84 years, with confidence intervals not including unity. In men, but not women, the association between work stress and higher risk of chronic disease appeared partially attributable to a higher number of risky health behaviours among employees in occupation with high prevalence of work stress. Supplementary analysis indicated robustness of the associations and showed a greater and statistically significant loss of chronic disease-free life years among women when increasing exposure contrast.

### Comparison with previous research studies

To our knowledge, only one study has previously investigated the association between work stress and chronic disease-free life expectancy. Magnusson Hanson et al. [11] reported that job strain was associated with a loss of 0.6 and 0.8 chronic disease-free life years (years free from cardiovascular disease, cancer, respiratory diseases and diabetes from age 50 to 75) in women and men, respectively, in a sample of 64,832 employees. In our analysis with a 25 times larger sample, a broader measure of work stress, including both job strain and ERI and the combination of these two stressors, and with a broader range of diseases we found a similar estimate for men and a slightly lower estimate for women. One important difference between the two studies is that Magnusson Hanson and colleagues measured job strain on the individual level, based on questionnaire responses, whereas we measured job strain and ERI based on a JEM. Thus, the measurement of work stressors by Magnusson Hanson et al. might have been less vulnerable to non-differential misclassification than our JEM-based measures but their measurement might have been more vulnerable to individual reporting bias than our measures that was based on job group and not on self-report. Furthermore, Magnusson Hanson et al. did not include ERI as a measure of psychosocial work stress in the analysis. Including ERI enabled us to demonstrate that the simultaneous exposure to both work stressors (job strain and ERI) was associated with higher number of years lost due to work stress than exposure to only one work stressor.

### Strength and limitations

The strengths of the present study are the large nationwide cohort, including all employees free of the studied chronic diseases in Denmark in the year 2000, aged 30–59. The use of occupational level information on work stress from JEMs reduced reporting bias, and the follow-up in nationwide health registers with no loss to follow-up. The applied broader exclusion criteria ensured that participants were not only free of the studied chronic diseases at baseline but also related diseases such as type-1 diabetes.

The generalisation of the study results might be limited to the examined group of employees in Denmark and we cannot rule out that associations might have been different in other age groups or countries. However, as results are based on a nationwide cohort of more than 1.5 million employees we expect to some degree our results to be generalisable to the workforce of European countries with similar workplace organisation as Denmark. Magnusson Hanson et al. [11] reported similar results for job strain among male employees from Finland, France, Sweden, and the UK, and we therefore feel confident that our results may be generalisable at least to the male workforce.

The JEMs enabled us to conduct analysis in a large nationwide cohort of more than 1.5 million employees in Denmark, which would be impossible with survey data. However, the method has some limitations. The use of JEMs can introduce exposure misclassification. As we use JEMs to assess the predicted prevalence of job strain and ERI, there may be individual-level variations within job groups, which we are not able to capture with the JEMs. Although the included JEMs are sex- and age-specific, they do not account for differences in the exposure level between individuals in the same job group. Some highly exposed individuals might be categorised as exposed and some as non-exposed depending on the average job group exposure. Contrary, some non-exposed individuals might be categorised as exposed if they were employed in a job group with high level of exposure on average. Hence, the “true” value for each individual will be random around the mean estimated job exposure value. We used a model-based approach to construct the JEM, which should yield, under certain assumptions, unbiased associations but with increased statistical uncertainty of the estimates (Berkson type error) [33, 34]. As the exposure misclassification is randomly assigned around the mean, and hence non-differential, our estimates might be biased towards the null [34]. The AUC for the job strain and the ERI JEM was 0.70 and 0.73, respectively, indicating that the JEM for these two exposures worked fairly well [35]. The intraclass correlation coefficients for the health behaviour JEMs, however, ranged from 0.26% for leisure time physical activity to 3.52% for smoking, indicating only small between-group variation and potential large misclassification. This was expected, but in the absence of any individual-level information of health behaviours, we chose this imperfect measure of the co-variate health behaviours over no measure of health behaviours, as suggested by Bondo Petersen et al. [25].

We assigned exposure to work stress once at baseline in year 2000 and kept this exposure constant throughout the 18 years of follow-up. As some employees may have changed jobs during follow-up, and thus changed exposure to work stress that we did not capture, there is a misclassification in the exposure that has likely biased results towards an underestimation of the role of work stress. Further studies examine the cumulative effect of work stress throughout the work life on chronic disease-free life expectancy are recommended.

In the present study, we included chronic diseases according to the World Health Organisation's priority of non-communicable chronic diseases target for prevention, supplemented with heart failure and dementia as done in previous research [1, 2, 12]. We did not include psychiatric disorders, such as depression and anxiety, in the outcome definition. Even though existing literature has found psychosocial work stressors, including job strain and ERI [36, 37], to be associated with psychiatric disorders, there is still uncertainty to whether such associations indicate a causal relation or could be explained by methodological bias [38]. We, therefore, did not want to mix physical diseases and psychiatric disorders in the same analysis. We encourage future research to investigate the association between work stress and psychiatric disorder-free life years. As psychiatric disorders tend to emerge in childhood and adolescence [39], a suitable cohort for this should as well include participants under the age of 30.

We found large heterogeneity in the incidence of the eight included chronic diseases (10% in first diagnose of cancer and 0.1% in first diagnose of dementia). Including diseases with large heterogeneity in incidences might affect the results if there are outcome-specific associations in different directions. However, among men outcome-specific analyses showed that in six out of eight analyses, there was a higher risk among participants with high prevalence of work stress compared to among participants in occupation with low prevalence. Among women, the outcome-specific analyses showed a less clear pattern, which is in line with the overall weaker association we found among women in the main analysis. Therefore, we judged it unlikely that variation in chronic disease incidence had affected the estimated years lost due to work stress among men. As we found less clear associations among women, we encourage future research to investigate sex differences in the association between work stress and specific chronic diseases.

We estimated chronic disease-free life years using adjusted survival curves. Previous studies have used different methods to estimate chronic disease-free life expectancy such as the SPACE (Stochastic Population Analysis of Complex Event) program [11] and bootstrapping methods to calculate confidence intervals [40]. A different methodological approach might produce different results. However, as covariate-specific analysis on socioeconomic position and risk health behaviours showed similar results (Supplementary material, Appendix-5, Table A-7) as previous studies [12, 40–43] we expect that such differences would be small.

## Conclusion

In an 18-year follow-up of the Danish workforce, work stress, measured as the combination of job strain and effort effort-reward imbalance, was associated with a slightly higher future incidence of chronic disease and with a small loss of years lived without chronic disease. The association was robust in men but inconclusive in women. In men, risky health behaviour might have been a part of the mechanism.

## Supplementary Information

Below is the link to the electronic supplementary material.

Supplementary file1 (DOCX 89 kb)

## Data Availability

All data are stored in a protected server environment hosted by Statistics Denmark and can be accessed by researchers registered with Statistics Denmark. For further information, please contact Professor Reiner Rugulies (rer@nfa.dk).
