# Supplementary material for: Work stress and loss of years lived without chronic disease: an 18-year follow-up of 1.5 million employees in Denmark
Source: Eur J Epidemiol. 2022 Mar 21;37(4):389–400. doi: 10.1007/s10654-022-00852-x (PMC9187572; doi:10.1007/s10654-022-00852-x)
Supplement: Supplementary file 1 — Supplementary file1 (DOCX 89 kb) [file 10654_2022_852_MOESM1_ESM.docx]

# Supplementary material for manuscript: Work stress and loss of years lived without chronic disease: An 18-year follow-up of 1.5 million employees in Denmark

### Appendix 1: Ascertainment of job strain and effort-reward imbalance with job exposure matrices

- Appendix 2: ICD-10 codes used for identifying incidence of the eight chronic diseases during follow-up
- Appendix 3: ICD-8 and ICD-10 codes used for identifying prevalence of the eight chronic diseases before start of follow-up

### Appendix 4: Description of covariates

- Appendix 5: Results from the supplementary analyses
- Reference

## Appendix 1: Ascertainment of job strain and effort-reward imbalance with job exposure matrices

To ascertain job strain and effort-reward imbalance (ERI) we constructed job exposure matrices (JEM) based on information from the Danish Work Environment Cohort Study (DWECS) and assigned it to individuals in the JEMPAD cohort. DWECS is a survey on working conditions and health conducted in a random sample of employed individuals in Denmark aged 18 to 64 years[1, 2] first drawn in 1990 and followed-up every fifth year until 2010, with inclusion of additional individuals in each wave. We included DWECS data from the 2000 and 2005 waves.

In accordance with previous research on job strain and cardiovascular disease using DWECS data,[3-5] we measured job strain by combining three items measuring job demands and five items measuring job control (see **table A-1**). Respondents were included if they responded to at least two of the three job demands items and to at least three of the five job control items (n=10,749). We calculated scales for job demands and job control with higher scores indicating higher job demands and higher job control, respectively. We defined job strain in DWECS respondents as scoring simultaneously above the median on job demands and below the median on job control, as in previous research. [3-5] In line with previous research,[6, 7] we defined ERI as the combination of four items on effort and four items on reward in DWECS (see **table A-1).** We included respondents if they responded to at least three of the four questions on both the effort and the reward scales (n=10,391). The effort and reward items were dichotomized (see **table A-1**) and sum scores ranging from 4 to 8 were calculated with higher scores indicating higher effort and higher reward, respectively. Missing values on items were treated as the mean of the three other effort and reward items, respectively. An effort-reward ratio was calculated as the effort score divided by the reward score. Finally, ERI was defined by dichotomizing the effort-reward ratio. Respondents with an effort-reward ratio > 1 were defined as having ERI, all other respondents were defined as not having ERI. Using multilevel modelling of the DWECS data, we constructed JEMs for the predicted probability of job strain and ERI given job group (coded according to DISCO-88, the Danish version of the International Standard Classification of Occupations (ISCO)-88 system)[8], sex, age, and year of data collection (2000, 2005), respectively. Job groups with four or less respondents on the four-digit level of DISCO-88 were collapsed with other similar small job groups at the three-digit, two-digit or one-digit classification level in the DISCO-88 classification system. Area under the curve (AUC) for the job exposure matrices was estimated with the ROC function in SAS with PROC logistics. AUC was 0.70 and 0.73 for job strain and ERI, respectively.

## Table A-1 Job strain and ERI questions from the Danish Work Environment Cohort Study

|  | Questions | Response | Dichotomisation |
| --- | --- | --- | --- |
| Psychological demands | Do you have to work very fast? | 1: Never/hardly ever;  2: Seldom;  3: Sometimes;  4: Often;  5: Always |  |
|  | How often do you not have time to complete all your work tasks? | 1: Never/hardly ever;  2: Seldom;  3: Sometimes;  4: Often;  5: Always |  |
|  | Contradictory demands are placed on you at work? | 1: Not correct;  2: From time to time correct;  3: Correct |  |
| Job control | Do you have any influence on what you do at work? | 1: Never/hardly ever;  2: Seldom;  3: Sometimes;  4: Often;  5: Always |  |
|  | Can you use your skills or expertise in your work? | 1: To a very small extent;  2: Not very much;  3: Somewhat;  4: To some extent;  5: To a very large extent |  |
|  | Do you have the possibility of learning new things through your work? | 1: To a very small extent;  2: Not very much;  3: Somewhat;  4: To some extent;  5: To a very large extent |  |
|  | Does your work require you to take the initiative? | 1: To a very small extent;  2: Not very much;  3: Somewhat;  4: To some extent;  5: To a very large extent |  |
|  | Is your work varied? | 1: To a very small extent;  2: Not very much;  3: Somewhat;  4: To some extent;  5: To a very large extent |  |
| Effort | How often do you not have time to complete all your work tasks? | 1: Never/hardly ever;  2: Seldom;  3: Sometimes;  4: Often;  5: Always | 1: Never/hardly ever, Seldom, Sometimes 2: Often, Always |
|  | Is your work unevenly distributed so it piles up? | 1: Never/hardly ever;  2: Seldom;  3: Sometimes;  4: Often;  5: Always | 1: Never/hardly ever, Seldom, Sometimes 2: Often, Always |
|  | Do you have to work overtime? | 1: Never/hardly ever;  2: Seldom;  3: Sometimes;  4: Often;  5: Always | 1: Never/hardly ever, Seldom, Sometimes 2: Often, Always |
|  | Do you have to work very fast? | 1: Never/hardly ever; 2: Seldom;  3: Sometimes;  4: Often;  5: Always | 1: Never/hardly ever, Seldom, Sometimes 2: Often, Always |
| Reward | Is your work recognized and appreciated by management? | 1: To a very small extent;  2: Not very much;  3: Somewhat;  4: To some extent;  5: To a very large extent | 1: To a very small extent, Not very much, Somewhat  2: To some extent, To a very large extent |
|  | Have you good prospects for the future in your job? | 1: To a very small extent;  2: Not very much;  3: Somewhat;  4: To some extent;  5: To a very large extent | 1: To a very small extent, Not very much, Somewhat  2: To some extent, To a very large extent |
|  | Are you worried about being transferred to another job against your will? | 1: Yes,  2: No | 1: Yes,  2: No |
|  | Are you worried about becoming unemployed? | 1: Yes,  2: No | 1: Yes,  2: No |

## Appendix 2: ICD-10 codes used for identifying incidence of the eight chronic diseases during follow-up

- Type 2 diabetes: E11
- Coronary heart disease (CHD): Either incident non-fatal myocardial infarction (I21, I22) or death due to CHD (I20 to I25)
- Stroke: I60, I61, I63, I64
- Cancers: C00 to C97
- Asthma: J45, J46
- Chronic Obstructive Pulmonary Disease (COPD): J41 to J44
- Heart failure: I50
- Dementia: Alzheimer's disease (F0, G30), vascular dementia (F01), dementia in other diseases classified elsewhere (F02, G31), unspecified dementia (F03).

**Appendix 3: ICD-8 and ICD-10 codes used for identifying prevalence of the eight chronic diseases before start of follow-up**

Note that ICD-9 never was used in Denmark.

- Type 1 and type 2 diabetes: ICD-8: 249, 250; ICD-10: E10, E11
- Coronary heart disease (CHD): ICD-8: 410 to 414; ICD-10: I20 to I25
- Stroke: ICD-8: 430 to 438; ICD-10: I60 to I69
- Cancers: ICD-8: 140 to 209; ICD-10: C00 to C97
- Asthma: ICD-8: 493; ICD-10: J45 to J46
- Chronic Obstructive Pulmonary Disease (COPD): ICD-8: 491 to 492; ICD-10: J41 to J44
- Heart failure: ICD-8: 427; ICD-10: I50
- Dementia: ICD-8: 290; ICD-10: F00 to F03, G30 to G31

## Appendix 4: Description of covariates

Information on migration background was retrieved from the Population Register.[9] We categorized migration background into ((i) Danish origin (the whole population in Denmark except immigrants and descendants of immigrants), (ii) immigrants (born abroad and none of the parents were either Danish citizens or born in Denmark), and (iii) descendants of immigrants (born in Denmark and none of the parents were either Danish citizens or born in Denmark)). Information on family type was retrieved by combining information from the Population Register[9] and the Family Relation Register[10] at Statistics Denmark. We defined family type in six groups based on the combination of cohabitation (single or married/cohabiting) and children living at home (single without children, single with children below age 8, single with children age 8 to 17 without children below age 8, married/cohabitant without children, married/cohabitant with children below age 8, or married/cohabitant with children age 8 to 17 without children below age 8). We obtained information on number of health services used, provided by primary health care professionals, such as general practitioners, from the Danish National Health Service Register[11] and categorized the number of health services used into quartiles based on the distribution within each year. Information on equivalent household disposable income accounting for household size was retrieved from the Danish registers on personal income and transfer payments[12] and categorized into quartiles based on the distribution the study population in the 2000.

We assigned JEMs for predicted probability of smoking and predicted levels of weekly alcohol intake, BMI and leisure time physical activity to each individual based on sex, age, period and job title (DISCO-88). We used the 1996 to 2000 period specific health behaviour JEMs from the Danish Occupational Cohort (DOC*X) study.[13] The construction of the JEMs are presented elsewhere.[13] Intraclass correlation coefficient (ICC) was 3.52%, 2.12%, 2.81%, and 0.26% for smoking, BMI, alcohol and leisure time physical activity, respectively. We calculated the number of risky health behaviours separately for women and men to account for overall sex differences in the JEMs. Based on the distributions of the predicted probability of smoking, predicted level of BMI, and the predicted level of weekly alcohol consumptions, we categorized individuals into high risk of smoking, high BMI, and high weekly alcohol consumption with cut-points at the highest tertile, respectively. Based on the distribution of the predicted level of leisure time physical activity, we categorized individuals intro low leisure time physical activity with a cut-point at the lowest tertile. We then calculated number of risky health behaviours as the sum of the four dichotomized variables (range 0 to 4). As few individuals experienced four risky health behaviours, we collapsed three and four risky health behaviours.

## Appendix 5: Results from the supplementary analyses

**Table A-2 Association of work stress with incident chronic disease, chronic-disease-free life expectancy from age 30 to 75 and years lost due to work stress among women and men with restricted outcome (type 2 diabetes, CHD, stroke, asthma, COPD and cancer only)**

|  |  | Hazard ratio (95% CI) for incident chronic disease | Chronic disease-free life expectancy (95% CI)* | Years lost due to work stress* |
| --- | --- | --- | --- | --- |
|  |  | **Model 2*** |  |  |
|  |  |  |  |  |
| Women (n=773,354) | |  |  |  |
|  | **Work stressors** |  |  |  |
|  | No stressors | 1.00 | 37.4 (37.2 to 37.6) | 0.00 |
|  | Job strain only | 1.04 (1.02 to 1.06) | 37.1 (36.9 to 37.3) | 0.25 (-0.11 to 0.61) |
|  | ERI only | 0.98 (0.97 to 1.00) | 37.5 (37.3 to 37.7) | -0.10 (-0.42 to 0.22) |
|  | Both stressors | 1.04 (1.02 to 1.05) | 37.1 (37.0 to 37.3) | 0.25 (-0.10 to 0.60) |
|  |  |  |  |  |
| Men (n=819,137) | |  |  |  |
|  | **Work stressors** |  |  |  |
|  | No stressors | 1.00 | 36.8 (36.7 to 36.9) | 0.00 |
|  | Job strain only | 1.01 (0.99 to 1.03) | 36.7 (36.6 to 36.9) | 0.05 (-0.23 to 0.33) |
|  | ERI only | 0.99 (0.99 to 1.00) | 36.8 (36.7 to 36.9) | -0.03 (-0.27 to 0.20) |
|  | Both stressors | 1.13 (1.11 to 1.14) | 35.9 (35.8 to 36.1) | 0.84 (0.57 to 1.11) |

* Incident chronic disease, chronic disease-free life expectancy from age 30 to 75 and years lost due to work stress adjusted for age (underlying time scale), migration background, family type, household disposable income, and number of health services used.

**Table A-3 Associations between work stress and incident chronic disease, chronic disease-free life expectancy from age 30 to 75 and years lost due to work stress among women and men with higher degree of exposure contrast (cut point for job strain = 10.7% and cut point for ERI = 23.8%)**

|  |  | Hazard ratio (95% CI) for incident chronic disease | Chronic disease-free life expectancy (95% CI)* | Years lost due to work stress* |
| --- | --- | --- | --- | --- |
|  |  | **Model 2*** |  |  |
|  |  |  |  |  |
| Women (n=773,354) | |  |  |  |
|  | **Work stressors** |  |  |  |
|  | No stressors | 1.00 | 37.3 (37.1 to 37.4) | 0.00 |
|  | Job strain only | 0.98 (0.96 to 1.00) | 37.4 (37.2 to 37.6) | -0.14 (-0.51 to 0.23) |
|  | ERI only | 0.98 (0.97 to 0.99) | 37.4 (37.2 to 37.6) | -0.13 (-0.46 to 0.19) |
|  | Both stressors | 1.06 (1.04 to 1.08) | 36.8 (36.6 to 37.1) | 0.41 (0.03 to 0.79) |
|  | |  |  |  |
| Men (n=819,137) | |  |  |  |
|  | **Work stressors** |  |  |  |
|  | No stressors | 1.00 | 36.4 (36.3 to 36.5) | 0.00 |
|  | Job strain only | 1.01 (0.99 to 1.03) | 36.4 (36.2 to 36.6) | 0.03 (-0.26 to 0.32) |
|  | ERI only | 0.98 (0.97 to 0.99) | 36.6 (36.5 to 36.7) | -0.16 (-0.40 to 0.09) |
|  | Both stressors | 1.12 (1.10 to 1.13) | 35.6 (35.5 to 35.8) | 0.80 (0.51 to 1.08) |

* Incident chronic disease, chronic disease-free life expectancy from age 30 to 75 and years lost due to work stress adjusted for age (underlying time scale), migration background, family type, household disposable income, and number of health services used.

**Table A-4 Association between work stress and incident chronic disease, chronic disease-free life expectancy from age 50 to 75 and years lost due to work stress among participants age 50 or above at baseline.**

|  |  | Hazard ratio (95% CI) for incident chronic disease | Chronic disease-free life expectancy (95% CI)* | Years lost due to work stress* |
| --- | --- | --- | --- | --- |
|  |  | **Model 2*** |  |  |
|  | |  |  |  |
| Women (n=222,826) | |  |  |  |
|  | **Work stressors** |  |  |  |
|  | No stressors | 1.00 | 20.3 (20.2 to 20.5) | 0.00 |
|  | Job strain only | 0.97 (0.93 to 1.01) | 20.5 (20.3 to 20.7) | -0.13 (-0.45 to 0.19) |
|  | ERI only | 1.00 (0.99 to 1.02) | 20.3 (20.2 to 20.5) | 0.00 (-0.25 to 0.26) |
|  | Both stressors | 1.04 (1.02 to 1.07) | 20.2 (20.0 to 20.3) | 0.18 (-0.11 to 0.46) |
| Men (n=238,315) | |  |  |  |
|  | **Work stressors** |  |  |  |
|  | No stressors | 1.00 | 19.3 (19.1 to 19.4) | 0.00 |
|  | Job strain only | 0.99 (0.96 to 1.02) | 19.3 (19.1 to 19.5) | -0.04 (-0.32 to 0.23) |
|  | ERI only | 1.01 (1.00 to 1.03) | 19.2 (19.1 to 19.3) | 0.06 (-0.16 to 0.28) |
|  | Both stressors | 1.10 (1.08 to 1.13) | 18.8 (18.6 to 18.9) | 0.48 (0.22 to 0.73) |

* Incident chronic disease, chronic disease-free life expectancy from age 50 to 75 and years lost due to work stress adjusted for age (underlying time scale), migration background, family type, household disposable income, and number of health services used.

**Table A-5 Association between job strain and effort-reward imbalance, analysed separately, and incident of chronic disease, chronic disease-free life expectancy from age 30 to 75 and years lost due to work stress among women and men**

|  |  | Hazard ratio (95% CI) for incident chronic disease | Chronic disease-free life expectancy (95% CI)* | Years lost due to work stress* |
| --- | --- | --- | --- | --- |
|  |  | **Model 2*** |  |  |
|  |  |  |  |  |
| Women (n=773,355) | |  |  |  |
|  | **Job strain** |  |  |  |
|  | No | 1.00 | 37.3 (37.2 to 37.5) | 0.00 |
|  | Yes | 1.04 (1.03 to 1.06) | 37.0 (36.9 to 37.2) | 0.28 (-0.05 to 0.61) |
|  | **ERI** |  |  |  |
|  | No | 1.00 | 37.3 (37.1 to 37.4) | 0.00 |
|  | Yes | 1.00 (0.99 to 1.01) | 37.3 (37.1 to 37.5) | -0.03 (-0.35 to 0.29) |
| Men (n=819,138) | |  |  |  |
|  | **Job strain** |  |  |  |
|  | No | 1.00 | 36.5 (36.4 to 36.6) | 0.00 |
|  | Yes | 1.08 (1.07 to 1.09) | 36.0 (35.8 to 36.1) | 0.54 (0.28 to 0.79) |
|  | **ERI** |  |  |  |
|  | No | 1.00 | 36.5 (36.4 to 36.6) | 0.00 |
|  | Yes | 1.02 (1.02 to 1.03) | 36.3 (36.2 to 36.5) | 0.17 (-0.07 to 0.41) |

* Incident chronic disease, chronic disease-free life expectancy from age 30 to 75 and years lost due to work stress adjusted for age (underlying time scale), migration background, family type, household disposable income, and number of health services used.

**Table A-6 Association between work stress and incident chronic disease by income in quartiles among women (n=773,355) and men (n=819,138)**

|  |  |  | Hazard ratio (95% CI) for cause specific incident of chronic disease |
| --- | --- | --- | --- |
|  |  |  | **Model 2*** |
|  |  |  |  |
| Women | | |  |
|  | **Low income** | No Stressors | 1.00 |
|  |  | Job strain only | 1.06 (1.02 to 1.09) |
|  |  | ERI only | 0.96 (0.94 to 0.99) |
|  |  | Both stressors | 1.02 (0.99 to 1.06) |
|  |  |  |  |
|  | **Medium-low income** | No Stressors | 1.00 |
|  |  | Job strain only | 1.04 (1.00 to 1.08) |
|  |  | ERI only | 0.96 (0.94 to 0.99) |
|  |  | Both stressors | 1.06 (1.03 to 1.10) |
|  |  |  |  |
|  | **Medium-high income** | No Stressors | 1.00 |
|  |  | Job strain only | 1.04 (1.00 to 1.08) |
|  |  | ERI only | 0.99 (0.97 to 1.01) |
|  |  | Both stressors | 1.05 (1.02 to 1.09) |
|  |  |  |  |
|  | **High income** | No Stressors | 1.00 |
|  |  | Job strain only | 1.00 (0.96 to 1.04) |
|  |  | ERI only | 1.00 (0.98 to 1.02) |
|  |  | Both stressors | 1.01 (0.98 to 1.05) |
|  |  |  |  |
| Men | |  |  |
|  | **Low income** | No Stressors | 1.00 |
|  |  | Job strain only | 0.97 (0.93 to 1.01) |
|  |  | ERI only | 1.00 (0.97 to 1.02) |
|  |  | Both stressors | 1.14 (1.10 to 1.17) |
|  |  |  |  |
|  | **Medium-low income** | No Stressors | 1.00 |
|  |  | Job strain only | 1.01 (0.98 to 1.05) |
|  |  | ERI only | 1.00 (0.98 to 1.02) |
|  |  | Both stressors | 1.12 (1.09 to 1.15) |
|  |  |  |  |
|  | **Medium-high income** | No Stressors | 1.00 |
|  |  | Job strain only | 1.01 (0.97 to 1.05) |
|  |  | ERI only | 0.99 (0.97 to 1.00) |
|  |  | Both stressors | 1.09 (1.06 to 1.12) |
|  |  |  |  |
|  | **High income** | No Stressors | 1.00 |
|  |  | Job strain only | 1.01 (0.97 to 1.06) |
|  |  | ERI only | 1.01 (0.99 to 1.02) |
|  |  | Both stressors | 1.11 (1.07 to 1.15) |
|  |  |  |  |

* Incident chronic disease adjusted for age (underlying time scale), migration background, family type, household disposable income, and number of health services used.

**Table A-7 Association between work stress and outcome-specific incident of the eight chronic diseases among women (n=773 355) and men (n=819 138)**

|  |  |  | Cases | Hazard ratio (95% CI) for cause specific incident of chronic disease |
| --- | --- | --- | --- | --- |
|  |  |  |  | **Model 2*** |
|  |  |  |  |  |
| Type 2 diabetes | | |  |  |
|  | **Women** | No Stressors | 11 605 | 1.00 |
|  |  | Job strain only | 4 679 | 1.15 (1.09 to 1.21) |
|  |  | ERI only | 1 667 | 0.94 (0.91 to 0.98) |
|  |  | Both stressors | 1 958 | 1.04 (0.99 to 1.09) |
|  |  |  |  |  |
|  | **Men** | No Stressors | 17 875 | 1.00 |
|  |  | Job strain only | 10 407 | 0.96 (0.92 to 1.01) |
|  |  | ERI only | 1 997 | 1.04 (1.01 to 1.07) |
|  |  | Both stressors | 3 722 | 1.17 (1.13 to 1.22) |
|  |  |  |  |  |
| Coronary heart disease (CHD) | | |  |  |
|  | **Women** | No Stressors | 4 714 | 1.00 |
|  |  | Job strain only | 2 003 | 1.12 (1.03 to 1.22) |
|  |  | ERI only | 644 | 0.93 (0.88 to 0.98) |
|  |  | Both stressors | 869 | 1.11 (1.03 to 1.20) |
|  |  |  |  |  |
|  | **Men** | No Stressors | 14 859 | 1.00 |
|  |  | Job strain only | 8 577 | 1.02 (0.97 to 1.08) |
|  |  | ERI only | 1 783 | 0.99 (0.96 to 1.01) |
|  |  | Both stressors | 3 062 | 1.15 (1.11 to 1.20) |
|  |  |  |  |  |
| Stroke | | |  |  |
|  | **Women** | No Stressors | 10 505 | 1.00 |
|  |  | Job strain only | 4 750 | 1.03 (0.97 to 1.09) |
|  |  | ERI only | 1 295 | 1.01 (0.97 to 1.04) |
|  |  | Both stressors | 1 746 | 1.05 (0.99 to 1.10) |
|  |  |  |  |  |
|  | **Men** | No Stressors | 14 792 | 1.00 |
|  |  | Job strain only | 9 153 | 1.09 (1.03 to 1.14) |
|  |  | ERI only | 1 864 | 0.99 (0.97 to 1.02) |
|  |  | Both stressors | 3 170 | 1.18 (1.13 to 1.22) |
|  |  |  |  |  |
| Cancers | | |  |  |
|  | **Women** | No Stressors | 50 469 | 1.00 |
|  |  | Job strain only | 23 823 | 1.01 (0.99 to 1.04) |
|  |  | ERI only | 6 125 | 1.01 (0.99 to 1.02) |
|  |  | Both stressors | 8 454 | 1.02 (1.00 to 1.05) |
|  |  |  |  |  |
|  | **Men** | No Stressors | 40 165 | 1.00 |
|  |  | Job strain only | 28 295 | 0.99 (0.96 to 1.02) |
|  |  | ERI only | 4 475 | 1.00 (0.98 to 1.01) |
|  |  | Both stressors | 7 413 | 1.03 (1.01 to 1.06) |
|  |  |  |  |  |
| Asthma | | |  |  |
|  | **Women** | No Stressors | 12 253 | 1.00 |
|  |  | Job strain only | 3 673 | 0.95 (0.90 to 1.00) |
|  |  | ERI only | 1 466 | 0.97 (0.93 to 1.01) |
|  |  | Both stressors | 1 537 | 0.95 (0.90 to 1.00) |
|  |  |  |  |  |
|  | **Men** | No Stressors | 8 027 | 1.00 |
|  |  | Job strain only | 2 940 | 0.97 (0.91 to 1.04) |
|  |  | ERI only | 898 | 0.92 (0.88 to 0.97) |
|  |  | Both stressors | 1 065 | 0.97 (0.91 to 1.04) |
|  |  |  |  |  |
| Chronic Obstructive Pulmonary Disease (COPD) | | |  |  |
|  | **Women** | No Stressors | 9 479 | 1.00 |
|  |  | Job strain only | 4 499 | 1.12 (1.05 to 1.19) |
|  |  | ERI only | 1 227 | 0.96 (0.92 to 0.99) |
|  |  | Both stressors | 1 902 | 1.16 (1.10 to 1.22) |
|  |  |  |  |  |
|  | **Men** | No Stressors | 8 838 | 1.00 |
|  |  | Job strain only | 5 605 | 1.07 (1.00 to 1.13) |
|  |  | ERI only | 1 085 | 0.97 (0.94 to 1.00) |
|  |  | Both stressors | 2 390 | 1.40 (1.33 to 1.46) |
|  |  |  |  |  |
| Heart failure | | |  |  |
|  | **Women** | No Stressors | 2 421 | 1.00 |
|  |  | Job strain only | 1 168 | 1.08 (0.95 to 1.22) |
|  |  | ERI only | 291 | 0.92 (0.86 to 0.99) |
|  |  | Both stressors | 400 | 1.00 (0.90 to 1.11) |
|  |  |  |  |  |
|  | **Men** | No Stressors | 6 107 | 1.00 |
|  |  | Job strain only | 4 055 | 0.98 (0.91 to 1.06) |
|  |  | ERI only | 687 | 1.01 (0.97 to 1.05) |
|  |  | Both stressors | 1 256 | 1.10 (1.03 to 1.16) |
|  |  |  |  |  |
| Dementia | | |  |  |
|  | **Women** | No Stressors | 348 | 1.00 |
|  |  | Job strain only | 276 | 0.94 (0.62 to 1.41) |
|  |  | ERI only | 25 | 1.13 (0.96 to 1.32) |
|  |  | Both stressors | 48 | 1.00 (0.74 to 1.35) |
|  |  |  |  |  |
|  | **Men** | No Stressors | 357 | 1.00 |
|  |  | Job strain only | 322 | 1.16 (0.83 to 1.61) |
|  |  | ERI only | 40 | 1.05 (0.90 to 1.22) |
|  |  | Both stressors | 74 | 1.14 (0.89 to 1.47) |
|  |  |  |  |  |

* Outcome specific incident of the eight chronic diseases, due to work stress adjusted for age (underlying time scale), migration background, family type, household disposable income, and number of health services used. The analyses are censored due to diagnoses or death of the seven other chronic diseases.

**Table A-8 Risk of incident chronic disease, chronic disease-free life expectancy and years lost due to work stress for the covariates in the study (sociodemographic characteristics, number of health service use and number of risky health behaviours).**

|  | Person-years | Cases | Cases per  1000 person-  years | Hazard ratio (95% CI) for incident chronic disease* | Chronic  disease-free  life expectancy  (95% CI)* | Years lost due  to work stress* |
| --- | --- | --- | --- | --- | --- | --- |
| Sex |  |  |  |  |  |  |
| Women | 12 283 514 | 176 319 | 14.4 | 1.00 | 37.1 (37.0 to 37.1) | 0.00 |
| Men | 12 608 508 | 215 359 | 17.1 | 1.22 (1.22 to 1.23) | 35.7 (35.6 to 35.8) | 1.38 (1.25 to 1.51) |
|  |  |  |  |  |  |  |
| Migration background |  |  |  |  |  |  |
| Danish origin | 3 837 937 | 375 869 | 15.8 | 1.00 | 37.1 (37.0 to 37.1) | 0.00 |
| Immigrant | 1 017 434 | 15 333 | 15.1 | 1.05 (1.04 to 1.07) | 36.7 (36.6 to 36.9) | 0.36 (0.17 to 0.55) |
| Descendant of immigrants | 36 651 | 476 | 13.0 | 0.98 (0.89 to 1.07) | 37.3 (36.6 to 37.9) | -0.16 (-0.84 to 0.51) |
|  |  |  |  |  |  |  |
| Family type |  |  |  |  |  |  |
| Single without children | 4 592 190 | 86 546 | 18.8 | 1.00 | 36.0 (35.9 to 36.0) | 0.00 |
| Single with children   (age 0 to 7) | 439 108 | 4053 | 9.2 | 0.92 (0.89 to 0.95) | 36.6 (36.4 to 36.8) | -0.63 (-0.86 to -0.40) |
| Single with children   (age 8 to 17) | 858 624 | 13 874 | 16.2 | 0.96 (0.94 to 0.97) | 36.3 (36.2 to 36.5) | -0.36 (-0.50 to -0.22) |
| Married/cohabitant  without children | 7 272 875 | 155 577 | 21.4 | 0.87 (0.86 to 0.88) | 37.0 (36.9 to 37.1) | -1.02 (-1.12 to -0.93) |
| Married/cohabitant  with children (age 0 to 7) | 3 964 469 | 30 409 | 7.7 | 0.74 (0.73 to 0.75) | 38.1 (38.0 to 38.2) | -2.12 (-2.21 to -2.03) |
| Married/cohabitant  with children (age 8 to 17) | 4 699 047 | 70 755 | 15.1 | 0.81 (0.80 to 0.82) | 37.5 (37.4 to 37.5) | -1.50 (-1.59 to -1.41) |
|  |  |  |  |  |  |  |
| Household disposable income |  |  |  |  |  |  |
| 1st (Lowest) Quartile | 5 906 665 | 83 963 | 14.2 | 1.28 (1.27 to 1.29) | 36.3 (36.2 to 36.4) | 1.69 (1.54 to 1.83) |
| 2nd Quartile | 5 927 563 | 86 206 | 14.5 | 1.16 (1.15 to 1.17) | 37.0 (36.2 to 36.4) | 1.00 (0.24 to 1.76) |
| 3rd Quartile | 5 826 959 | 97 058 | 16.7 | 1.10 (1.10 to 1.11) | 37.3 (36.9 to 37.0) | 0.65 (0.22 to 1.07) |
| 4th (Highest) Quartile | 5 733 315 | 103 603 | 18.1 | 1.00 | 38.0 (37.9 to 38.0) | 0.00 |
|  |  |  |  |  |  |  |
| Number of health services used |  |  |  |  |  |  |
| 1st (Lowest) Quartile | 5 568 291 | 80 719 | 14.5 | 1.00 | 37.8 (37.7 to 37.9) | 0.00 |
| 2nd Quartile | 6 688 963 | 94 370 | 14.1 | 0.95 (0.94 to 0.96) | 38.1 (38.1 to 38.2) | -0.32 (-0.45 to -0.18) |
| 3rd Quartile | 6 723 965 | 105 519 | 15.7 | 1.08 (1.07 to 1.09) | 37.3 (37.2 to 37.4) | 0.51 (0.37 to 0.65) |
| 4th (Highest) Quartile | 5 910 803 | 111 070 | 18.8 | 1.32 (1.31 to 1.33) | 35.8 (35.8 to 35.9) | 1.96 (1.81 to 2.11) |
|  |  |  |  |  |  |  |
| Number of risky  health behaviours |  |  |  |  |  |  |
| No risky health behaviours | 4 029 145 | 59 172 | 14.7 | 1.00 | 37.5 (37.5 to 37.6) | 0.00 |
| One risky health behaviours | 10 696 688 | 157 592 | 14.7 | 1.00 (0.99 to 1.01) | 37.5 (37.5 to 37.6) | -0.02 (-0.15 to 0.11) |
| Two risky health behaviours | 4 548 852 | 72 259 | 15.9 | 1.15 (1.13 to 1.16) | 36.6 (36.5 to 36.7) | 0.94 (0.78 to 1.09) |
| Three or four   risky health behaviours | 3 401 684 | 65 215 | 19.2 | 1.25 (1.24 to 1.27) | 35.9 (35.8 to 36.0) | 1.62 (1.46 to 1.78) |

* Incident chronic disease, chronic disease-free life expectancy from age 30 to 75 and years lost due to work stress adjusted for age (underlying time scale) and sex

References

1. Burr H, Bjorner JB, Kristensen TS, Tüchsen F, Bach E. Trends in the Danish work environment in 1990-2000 and their associations with labor-force changes. Scand J Work Environ Health. 2003;29(4):270-9.

2. Feveile H, Olsen O, Burr H, Bach E. Danish Work Environment Cohort Study 2005: From idea to sampling design. Statistics in Transition. 2007;8(3):441-58.

3. Fransson EI, Nyberg ST, Heikkilä K, Alfredsson L, De Bacquer D, Batty GD, et al. Comparison of alternative versions of the job demand-control scales in 17 European cohort studies: the IPD-Work consortium. BMC Public Health. 2012;12(1):62.

4. Kivimäki M, Nyberg ST, Batty GD, Fransson EI, Heikkilä K, Alfredsson L, et al. Job strain as a risk factor for coronary heart disease: a collaborative meta-analysis of individual participant data. Lancet. 2012;380(9852):1491-7.

5. Fransson EI, Nyberg ST, Heikkilä K, Alfredsson L, Bjorner JB, Borritz M, et al. Job strain and the risk of stroke: an individual-participant data meta-analysis. Stroke. 2015;46(2):557-9.

6. Siegrist J, Dragano N, Nyberg ST, Lunau T, Alfredsson L, Erbel R, et al. Validating abbreviated measures of effort-reward imbalance at work in European cohort studies: the IPD-Work consortium. International archives of occupational and environmental health. 2014;87(3):249-56.

7. Dragano N, Siegrist J, Nyberg ST, Lunau T, Fransson EI, Alfredsson L, et al. Effort-reward imbalance at work and incident coronary heart disease: a multi-cohort study of 90,164 individuals. Epidemiology. 2017.

8. International Labour Organization (ILO). ISCO-88. 2004 [Available from: <http://www.ilo.org/public/english/bureau/stat/isco/isco88/>.

9. Statistics Denmark. Documentation of statistics: Population and elections 2019 [Available from: <https://www.dst.dk/en/Statistik/dokumentation/documentationofstatistics?subject=02>.

10. Statistics Denmark. Documentation of statistics: Households, families and children 2019 [Available from: <https://www.dst.dk/en/Statistik/dokumentation/documentationofstatistics/households--families-and-children>.

11. Andersen JS, Olivarius Nde F, Krasnik A. The Danish National Health Service Register. Scandinavian journal of public health. 2011;39(7 Suppl):34-7.

12. Baadsgaard M, Quitzau J. Danish registers on personal income and transfer payments. Scandinavian journal of public health. 2011;39(7 Suppl):103-5.

13. Bondo Petersen S, Flachs EM, Prescott EIB, Tjønneland A, Osler M, Andersen I, et al. Job-exposure matrices addressing lifestyle to be applied in register-based occupational health studies. Occup Environ Med. 2018.
